# Endoscopic treatment of odontogenic cyst with intra-sinusal extension

**DOI:** 10.1016/S1808-8694(15)31343-4

**Published:** 2015-10-20

**Authors:** Antonio C. Cedin, Fausto A. de Paula, Emanuel R. Landim, Flávio L.P. da Silva, Luis F. de Oliveira, Ana C. Sotter

**Affiliations:** ^1^ Physician and coordinator, Service of Otorhinolaryngology, Hospital Beneficência Portuguesa de São Paulo; ^2^ Resident Physician, Service of Otorhinolaryngology, Hospital Beneficência Portuguesa de São Paulo

**Keywords:** odontogenic cyst, videoendoscopic surgery, oroantral fistula, chronic sinusitis

## Abstract

**O**dontogenic cyst is a common lesion that can happen after inflammation of the dental pulp. The therapeutic approach of these cysts is made at dentist's offices, and depending on their extension, they may develop oroantral fistula and chronic sinusitis. The objective of this study is to propose the videoendoscopic treatment of the odontogenic cyst with expression in the maxillary sinus. We made a retrospective study of four cases of cysts of dental origin, with intra-sinusal extension, complicated with oroantral fistula and chronic sinusitis of maxillary sinus after curettage in a dentist's office. We used the videoendoscopic technique through transmaxillary approach to access the intra-sinusal cyst. All the four patients presented resolution of the infectious manifestation and healing of the oroantral fistula, without recurrence within two years of follow-up. Videoendoscopic surgery is a safe and effective method for the management of odontogenic cysts with extension to maxillary sinus, and it may prevent oroantral fistula formation and chronic sinusitis.

## INTRODUCTION

Odontogenic cysts are lesions that frequently rise in the maxilla and/or mandible, originating from epithelial remains associated with odontogenesis. The most frequent types are: periapical cyst (65%), dentigerous cyst (24%) and primordial or keratocyst (5 a 8%).1., 2.

The majority of these cysts are managed at dentist's offices using surgical approaches such as enucleation with use of Milton's solution or combined with cryotherapy, simple enucleation, curettage, marsupialization and tooth extraction.

Intervention of cysts found in the maxillary sinus may lead to oroantral fistula formation and chronic rhinosinusitis. This study aims at proposing a videoendoscopic technique to treat odontogenic cyst with intrasinusal extension as a complementary approach of the odontological treatment.

## MATERIAL AND METHOD

**Figure 1:**
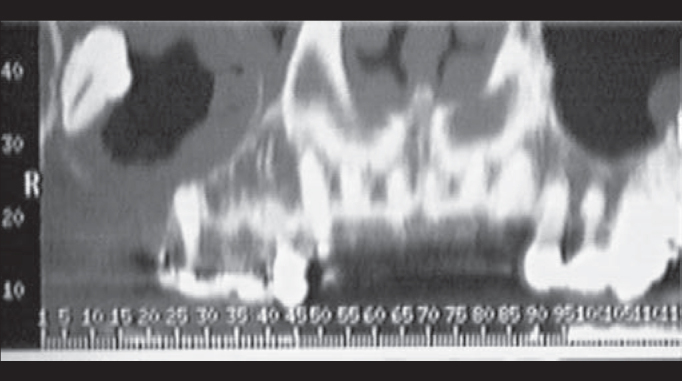
Computed tomography: Right intramaxillary cyst with oroantral fistula.

**Figure 2:**
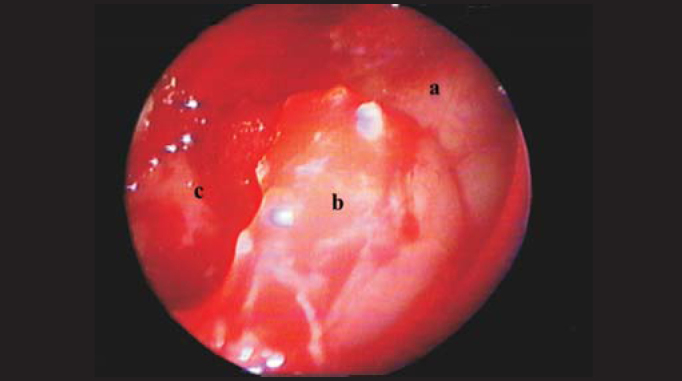
a: Posterior wall of maxillary antrum. b: Cyst capsule. c: Inner portion of cyst.

**Figure 3:**
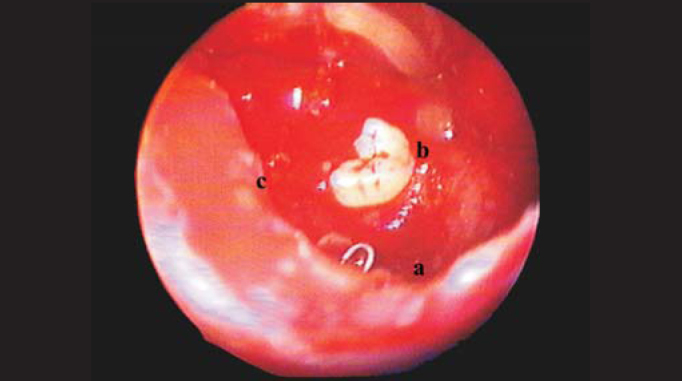
Maxillary sinus after cyst removal. a: Curette showing site of fistula. b: Included tooth. c: Lower edge of antrostomy.

Four cases of dental cysts with intrasinusal extension were operated on (Figures 1 and 2). They were outpatients treated by dentists who chose tooth extraction and transalveolar curettage as alternatives, which resulted in oroantral fistula (Figure 3) and chronic maxillary sinus suppuration.

**Figure 4:**
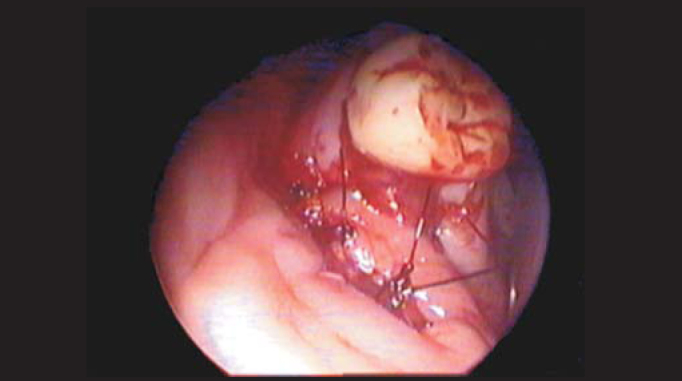
Oral aspect of mucous flap rotation over the oroantral fistula.

Patients were surgically treated under general anesthesia. The procedure of choice was combined access through the canine fossa with lower meatotomy. Such approach is performed by means of a 10mm-circumferential antrotomy to allow simultaneous management of the optical fiber (4mm-rigid endoscope at 0º, 30º e 70º) and of small clamps (forceps and curettes for sinusal endoscopic surgery), achieving cyst resection. The cyst should be removed in a way that only the bone bed remains on the sinusal wall at the implantation spot. In case of an oroantral fistula, curettage of the borders is also done. Occlusion is performed with a septal or conchal bone fragment as well as a flap rotation of the jugal or palatine mucosa (Figure 4) sutured with 3.0-mononylon thread.

## RESULTS

Infections of all four patients were resolved and fistula healing was reached. No relapses were reported after 2 years of follow-up. Cyst fragments obtained during surgery were sent for clinical pathology analysis and the results were compatible to periapical cyst. No signs of malignancy were found in the analyzed cases.

## DISCUSSION

A cyst is defined as a cavity layered by epithelium hosting liquid or semi-solid material originating from embryonic epithelial tissue. The epithelium of the odontogenic cyst may come from the following structures:

a) Epithelial fragments of the teeth crown.
b) Epithelial debris of Malassez (cells remains by development of teeth roots from inside of periodontal ligament)
c) Epithelial debris of Seres (remains of teeth lamina)
d) Fragments of teeth germ cells, including enamel, dental papilla and dental sac.

The most accepted hypothesis for cyst development is proliferation of epithelial fragments with formation of islets. Due to its nonvascular aspect, degeneration occurs centrally. In addition, as it is distant from the adjacent connective tissue, release of enzymes leads to degeneration of its own cell protoplasm, and dead cells turn into liquid material. The intracystic liquid has higher osmotic pressure than that of surrounding tissues, which leads to progressive cyst growth.

The dentigerous cyst occurs after development of the tooth crown with accumulation of liquid between the enamel epithelium and the tooth crown, directly linked to an unerupted tooth. This lesion frequently occurs in maxillary canine teeth and third lower molar teeth^3^. It is the most aggressive odontogenic cyst and it may reach large volumes as well as bulging of bone cortical structures.

Radiographically, this structure is characterized by a well-defined radiolucent image of bone cortical, involving an unerupted tooth crown and cervical onset. In order to differ from a dental sac image, the x-ray image should not be smaller than 2.5mm of extension. It may be central, lateral, circumferential or eruptional^4^.

Cell degeneration of the enamel's starry reticulum gives rise to the primary cyst before the mineralized tissue is formed. This cyst appears in place of a normal, unerupted or supra-numerous tooth^5^. It is frequently observed among young people, around the second decade of life. In absence of bulging, they are not perceptible and, in the great majority of cases, they are detected by x-ray only^6^. The posterior portion of the mandible is the region of preference. Edema, drainage, pain and infection may occur in 50% of the cases^7^. The image appears as a radio-opaque ring, sometimes presenting multinodular images^8^. According to Main^9^, the x-ray images may be classified as of reposition, involvement, extraneous or collateral.

The periapical cyst, the most common type, is the result of an inflammatory stimulus promoted by the dental pulp. In general, they are asymptomatic, detected by x-ray, and occur in the third or fourth decade of life. They appear as a radiolucent image of homogenous, oval or round density, which is related to an intact radicular apex with rupture of the hard layer and a radio-opaque bone sclerosis lining^10^. Generally, periapical cysts are small and treated by the dentist with periodontal approach and cyst enucleation, using Milton's solution. Generally, this approach leads to cyst involution. If cyst regression does not occur, an apicoectomy of the tooth root followed by curettage is done. In these cases, we suggest complementary endoscopic otolaryngology intervention. The approach consists of cyst exeresis by transmaxillary access and lower meatotomy for temporary drainage of the maxillary antrum. The oroantral fistula resulting from tooth extraction is simultaneously arranged with border curettage and autologous bone graft (septal, conchal or cortical of mastoid) with palatal or jugal mucous flap rotation. This procedure prevents the occurrence of oroantral fistula and sinusal suppuration, assuring complete cyst exeresis.

## CONCLUSION

We observed that the videoendoscopic surgery is a feasible alternative to treat an odontogenic cyst with maxillary sinusal extension, which may be adopted simultaneously to prevent sinusal suppuration and complete cyst exeresis.

Correction of oroantral fistulas caused by teeth extractions should be done by means of autologous bone graft and mucous flap rotation with suture.
